# Temporal and spatial dynamics of immune cells in spontaneous liver transplant tolerance

**DOI:** 10.1016/j.isci.2023.107691

**Published:** 2023-08-19

**Authors:** Weitao Que, Hisashi Ueta, Xin Hu, Miwa Morita-Nakagawa, Masayuki Fujino, Daisuke Ueda, Nobuko Tokuda, Wenxin Huang, Wen-Zhi Guo, Lin Zhong, Xiao-Kang Li

**Affiliations:** 1 Department of Hepatobiliary and Pancreatic Surgery, The First Affiliated Hospital of Zhengzhou University, Zhengzhou 450052, China; 2 Division of Transplantation Immunology, National Research Institute for Child Health and Development, Tokyo 157-8535, Japan; 3 Department of General Surgery, Shanghai General Hospital, Shanghai Jiao Tong University School of Medicine, Shanghai 200080, China; 4 Department of Anatomy, Dokkyo Medical University, Tochigi 321-0293, Japan; 5 Oral Medicine Research Center, Fukuoka Dental College, Fukuoka 814-0175, Japan; 6 Management Department of Biosafety, Laboratory Animal, and Pathogen Bank, National Institute of Infectious Diseases, Tokyo 162-8640, Japan; 7 Division of Hepato-Pancreato-Biliary Surgery and Transplantation, Department of Surgery, Kyoto University Graduate School of Medicine, Kyoto 606-8303, Japan

**Keywords:** Immunology, Cell biology, Organizational aspects of cell biology

## Abstract

The liver has long been deemed a tolerogenic organ. We employed high-dimensional mass cytometry and immunohistochemistry to depict the temporal and spatial dynamics of immune cells in the spleen and liver in a murine model of spontaneous liver allograft acceptance. We depicted the immune landscape of spontaneous liver tolerance throughout the rejection and acceptance stages after liver transplantation and highlighted several points of importance. Of note, the CD4^+^/CD8^+^ T cell ratio remained low, even in the tolerance phase. Furthermore, a PhenoGraph clustering analysis revealed that exhausted CD8^+^ T cells were the most dominant metacluster in graft-infiltrating lymphocytes (GILs), which highly expressed the costimulatory molecule CD86. The temporal and spatial dynamics of immune cells revealed by high-dimensional analyses enable a fine-grained analysis of GIL subsets, contribute to new insights for the discovery of immunological mechanisms of liver tolerance, and provide potential ways to achieve clinical operational tolerance after liver transplantation.

## Highlights

• Mass cytometry depicted the immune landscape of spontaneous liver tolerance
• CD8^+^ T cells persisted in high frequency in tolerant liver allografts
• The graft-infiltrating CD8^+^ T cells exhibited an exhausted phenotype
• CD86 costimulatory molecule were highly expressed by graft-infiltrating CD8^+^ T cells

## Graphical abstract

**Figure:**
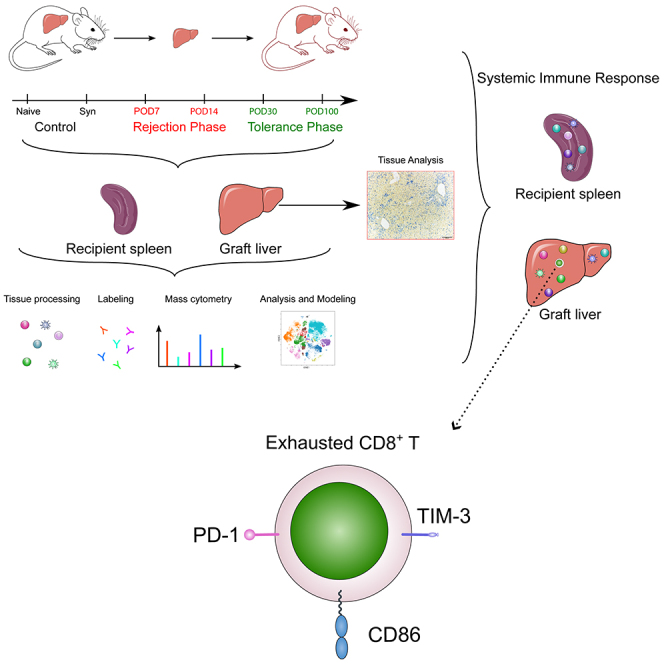

## Introduction

Liver transplantation is the optimal and major therapeutic approach for various end-stage liver diseases. With advances in surgical techniques, organ preservation, and immunosuppressive agents, the short-term outcomes after transplantation have greatly improved. However, life-long, systemic immunosuppressive treatment after transplantation is required to prevent rejection and graft loss. Such treatment is associated with severe side effects, including infection, renal injury, *de novo* cancer, and dysmetabolic syndrome. Achieving transplantation tolerance has long been the ultimate goal in the field of liver transplantation.

The liver has been deemed a lymphoid organ with a unique constituency of immune cells and exhibits inherent tolerogenic properties.^1^^,^^2^ Liver allografts are spontaneously accepted in multiple animal models without the need for immunosuppressive therapy. Liver transplant recipients require less immunosuppression than recipients of other organs. Operational tolerance also occurs in approximately 50% of carefully selected human liver allograft recipients who are intentionally weaned off all immunosuppressive agents.^3^^,^^4^ However, the immunological mechanisms underlying liver tolerance are poorly understood and remain the subject of active investigation.

Surgically demanding mouse liver transplantation is a valuable model for studying transplantation immunobiology.^5^^,^^6^ This model spontaneously accepts major histocompatibility complex (MHC)-mismatched liver allografts without immunosuppressive treatment. The spontaneously accepted liver allografts can induce subsequent tolerance to other transplanted organs from the same donor strain while rejecting third-party grafts.^7^ Liver transplantation can even reverse ongoing graft rejection of a previously transplanted organ in a donor-specific manner.^8^ Much effort has been made to uncover the cellular and molecular mechanisms of allograft tolerance, with a particular focus on the roles of donor-derived tissue cells or host immune cells.^5^^,^^9^ Morita et al. highlighted the importance of the programmed cell death protein 1 (PD-1)/B7-H1 pathway in the spontaneous acceptance of liver allografts.^10^^,^^11^ Ono et al. identified that graft-infiltrating programmed death ligand 1 (PD-L1)^hi^ cross-dressed dendritic cells (CD-DCs), which display intact donor MHC molecules, regulate anti-donor T cell responses in mouse liver transplant tolerance.^12^ An understanding of the mechanisms underlying liver allograft tolerance is crucial to developing more rationally designed treatment strategies for allograft rejection and autoimmune disease.

Mass cytometry or CyTOF (cytometry by time-of-flight), is a high-dimensional single-cell technology that enables simultaneous characterization of the immune system, provides the opportunity to make great advances in the scientific understanding of liver tolerance, and may demonstrate ways through which clinical operational tolerance after liver transplantation may potentially be achieved. The purpose of this study is to depict the immune landscape of spontaneous liver tolerance throughout the rejection and acceptance stages after liver transplantation and investigate the mechanisms underlying this tolerance.

## Results

### Subjects and study design

**Figure 1:**
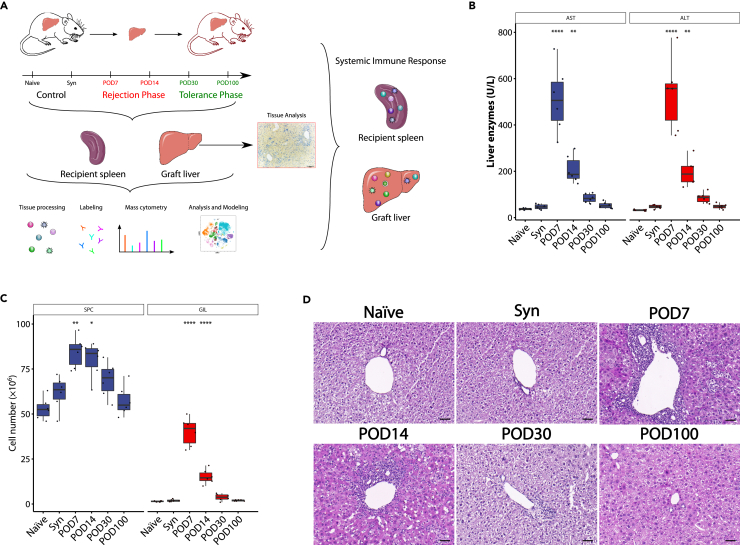
The phenotype of rejection activity after liver transplantation (A) Experiment design. High-dimensional mass cytometry and immunohistochemistry were conducted to depict the temporal and spatial dynamics of immune cells in mouse liver transplantation. B6/N liver allografts were transplanted into C3H mice. Total immune cells were isolated from spleen and liver grafts and then subjected to mass cytometry analysis on postoperative days (POD) 7, 14, 30, and 100. Liver graft tissue was used for immunohistochemistry. Syngeneic liver transplantation (C3H → C3H) mice were sampled on POD14 as a model control, and Naïve mice were sampled as a blank control (n = 3 for each group). (B) The serum liver enzyme levels in Naïve control (Naïve), syngeneic control (Syn), POD7, POD14, POD30, and POD100 groups (n = 6 for each group). ∗∗p < 0.01 and ∗∗∗∗p < 0.0001 by one-way ANOVA with Tukey’s post hoc test vs. syngeneic model control. Values are shown as the mean ± SEM. (C) The number of spleen cells (SPCs) and graft-infiltrating lymphocytes (GILs) in Naïve, Syn, POD7, POD14, POD30, and POD100 groups (n = 6 for each group). ∗p < 0.05, ∗∗p < 0.01, and ∗∗∗∗p < 0.0001 by one-way ANOVA with Tukey’s post hoc test vs. syngeneic model control. Values are shown as the mean ± SEM. (D) Representative H&E staining of liver grafts in Naïve, Syn, POD7, POD14, POD30, and POD100 groups. Image data are representative of six independent experiments. Scale bar = 50 μm.

To depict the temporal and spatial dynamics of major immune cells in mouse liver transplantation, we established a fully MHC-mismatched mouse liver transplantation model from C57BL/6N (B6/N; H-2k^b^) to C3H/HeNSlc (C3H; H-2k^k^) recipients (Figure 1A). A transient, self-refractory rejection episode occurred around postoperative day (POD) 7. All of the B6/N livers transplanted into the C3H mice were accepted and exhibited indefinite survival (median survival time >100 days). Spleen cells (SPCs) and liver graft-infiltrating lymphocytes (GILs) were examined by mass cytometry on POD7, POD14, POD30, and POD100. Syngeneic liver transplantation (C3H → C3H) mice were sampled on POD14 as a model control, and Naïve mice were sampled as a blank control. Liver graft tissue was used for immunohistochemistry. Other corresponding samples were also collected for further analyses.

The rejection activity was determined by serum liver enzyme levels, infiltrating immune cells, and H&E staining. After transplantation, the graft damage peaked on POD7, and then obviously and quickly declined indicated by serum liver enzyme levels (Figure 1B). Vigorous expansion of both SPCs and GILs suggested potent anti-donor immune responses after liver allograft implantation. The GILs gradually reduced and diminished spontaneously from the peak on POD7 (Figure 1C). An allograft histological analysis by H&E staining revealed severe hepatic parenchymal damage and inflammatory infiltration in the portal vein, bile duct, and hepatic vein on POD7 and POD14, which was relieved to moderate grade on POD30, and restored to normal liver architectures on POD100 (Figure 1D). Consistently, according to the Banff schema for grading and staging of liver allograft rejection, the rejection activity index (RAI) exhibited a similar trend with the aforementioned findings (Figure S1). All indices returned to within the normal levels on POD100, without the use of immunosuppressive drugs.

### Temporal dynamics of major immune cells after mouse liver transplantation

**Figure 2:**
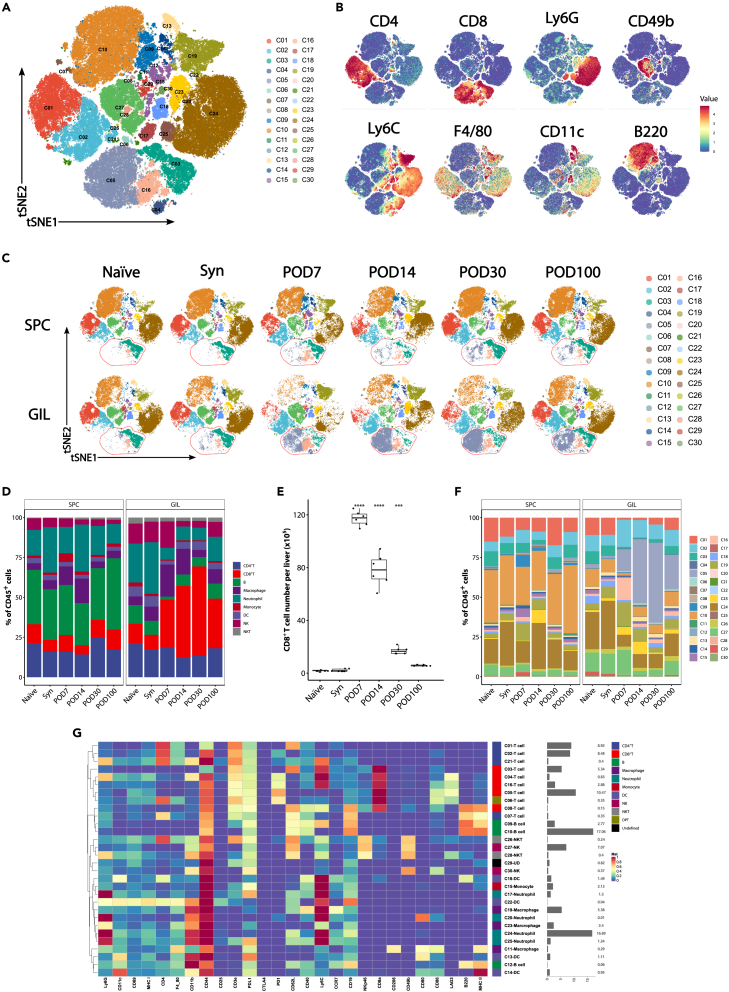
FitSNE analysis of major immune cells in SPCs and GILs (A) t-SNE map displaying immune cells pooled from 1,800,000 randomly selected cells (50,000 per file) in each individual sample. The FitSNE analysis generated clouds of cells. Each dot in the plots represents a single cell. Cells are colored by PhenoGraph cluster. (B) Normalized expression of major lineage markers on the t-SNE map. (C) t-SNE plot of CD45^+^ leukocytes, down-sample to 50,000 cells in each sample, in SPCs and GILs of Naïve control (Naïve), syngeneic control (Syn), POD7, POD14, POD30, and POD100 groups. Cells are colored by PhenoGraph cluster. The red dotted circles indicated CD8^+^ T cell islands. Data are representative of three independent experiments. (D) Stacked bar graph representing the proportions of major immune cell populations among CD45^+^ SPCs and GILs of Naïve Syn, POD7, POD14, POD30, and POD100 groups (n = 3 for each group). Values are shown as the mean. (E) The number of CD8^+^ T cells in GILs per liver graft in Naïve, Syn, POD7, POD14, POD30, and POD100 groups (n = 6 for each group). The cell numbers were calculated by multiplying the total GIL numbers to the proportions of CD8^+^ T cell of each graft. ∗∗∗p < 0.001 and ∗∗∗∗p < 0.0001 by one-way ANOVA with Tukey’s post hoc test vs. syngeneic model control. Values are shown as the mean ± SEM. (F) Stacked bar graph representing the proportions of PhenoGraph metaclusters among CD45^+^ SPCs and GILs of Naïve, Syn, POD7, POD14, POD30, and POD100 groups (n = 3 for each group). Values are shown as the mean. (G) Heatmap of the molecular marker expression of 30 metaclusters identified with PhenoGraph. Relative frequencies are displayed as a bar graph to the right.

To evaluate the temporal dynamics of major immune cells during the rejection and acceptance of a liver allograft after mouse liver transplantation, SPCs and GILs were probed with a panel of 29 metal-conjugated antibodies (Table 1) using a mass cytometer. We performed dimensionality reduction algorithm Fast interpolation-based t-distributed stochastic neighbor embedding (FitSNE) analysis and PhenoGraph clustering algorithm of single live CD45^+^ events to reveal distinct clustering of major immune cell types across the groups (Figure 2A). FitSNE maps were generated and colored by a variety of lineage markers (Figure 2B). From the FitSNE maps, we noted that the pattern of CD8^+^ T cells distinctly shifted over time in the GILs but not in the SPCs (Figure 2C**, bottom islands in red dotted circles)**. On the basis of marker expression, we manually gated 9 broad populations of leukocytes, including neutrophils, macrophages, monocytes, dendritic cells (DCs), natural killer (NK) cells, natural killer T (NKT) cells, B cells, CD4^+^ T cells, and CD8^+^ T cells, among which CD8^+^ T cells were the most dominant cell population in GILs after mouse liver transplantation (Figure 2D). The number of CD8^+^ T cells rapidly increased in the rejection phase and became the largest population in the transplanted liver allograft. On POD30, it accounted for over 50% of the total CD45^+^ cells, and the proportion remained high until POD100. However, the absolute quantity of CD8^+^ T cells per liver peaked on POD7 and declined thereafter (Figure 2E).

**Table 1:** List of metal-labeled antibodies used for mass cytometry

Reagents and antibodies	clone	label	product_id
Anti-CD45	30-F11	89Y	3089005C
Anti-Ly-6G	1A8	141Pr	3141008C
Anti-CD11c	N418	142ND	3142003C
Anti-CD69	H1.2F3	143ND	3143004C
Anti-MHC Class I	28-14-8	144ND	3144016C
Anti-CD4	RM4-5	145ND	3145002C
Anti-F4/80	BM8	146ND	3146008C
Anti-CD11b (Mac-1)	M1/70	148ND	3148003C
Anti-CD44	IM7	150ND	3150018C
Anti-CD25 (IL-2R)	3C7	151Eu	3151007C
Anti-CD3e	145-2C11	152Sm	3152004C
Anti-CD274 (PD-L1)	10F.9G2	153Eu	3153016C
Anti-CD152 (CTLA-4)	UC10-4B9	154Sm	3154008C
Anti-CD279 (PD-1)	29F.1A12	159Tb	3159024C
Anti-CD62L (L-selectin)	MEL-14	160Gd	3160008C
Anti-CD40	HM40-3	161Dy	3161020C
Anti-Ly-6C	HK1.4	162Dy	3162014C
Anti-CD197 (CCR7)	4B12	164Dy	3164013C
Anti-CD19	6D5	166Er	3166015C
Anti-CD335 (NKp46)	29A1.4	167Er	3167008C
Anti-CD8α	53–6.7	168Er	3168003C
Anti-CD206 (MMR)	C068C2	169Tm	3169021C
Anti-CD49b	HMa2	170Er	3170008C
Anti-CD80 (B7-1)	16-10A1	171Yb	3171008C
Anti-CD86	GL1	172Yb	3172016C
Anti-CD223 (LAG-3)	C9B7W	174Yb	3174019C
Anti-B220	RA3-6B2	176Yb	3176002C
Anti-I-A/I-E	M5/114.15.2	209Bi	3209006C
Cell ID Cisplatin 198Pt	N/A	198Pt	201198
Cell-ID™ DNA Intercalator-Ir	N/A	191Ir	201192A

The PhenoGraph clustering analysis identified 30 metaclusters overlaid on the FitSNE map including ten CD3^+^ T cell subsets, three B cell subsets, four DC subsets, four neutrophil subsets, three macrophage subsets, one monocyte subset, two NK subsets, two NKT subsets, and one undefined subset. (Figure 2C). The frequencies of each metacluster are illustrated in a barplot (Figure 2F). The marker expression profile of immune cell clusters was visualized in a heatmap (Figure 2G). Interestingly, a CD8^+^ T cell metacluster (metacluster C05) was present in tolerant grafts in high frequency but almost absent in SPCs and rejecting grafts (Figure 2F). One CD8^+^ T cell metacluster (metacluster C16) was predominantly enriched in rejecting grafts. One B cell metacluster (metacluster C09) was increased in the SPCs of a rejecting mouse, whereas another B cell metacluster (metacluster C10) was decreased in the SPCs and GILs of a rejecting mouse. One NK metacluster (metacluster C27) was reduced in tolerant grafts.

### Spatial dynamics of major immune cells after mouse liver transplantation

**Figure 3:**
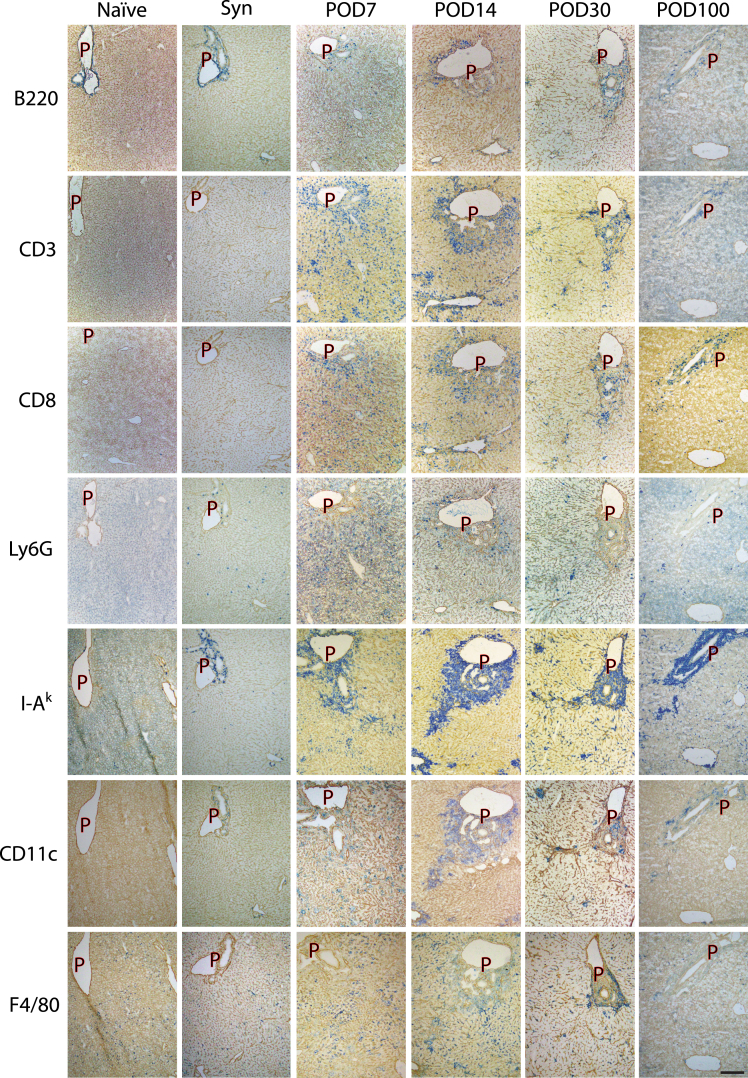
Immunohistochemical staining of different cell markers in hepatic grafts Different immune cell markers (B220, CD3, CD8, Ly6G, I-A^k^, CD11c, and F4/80) were stained in hepatic grafts in Naïve control (Naïve), syngeneic control (Syn), postoperative days (POD) 7, POD14, POD30, and POD100 groups. Image data are representative of three independent experiments. Scale bar = 200 μm.

We further identified the location of each major immune cell lineage by immunohistochemistry. GILs gradually increased, reaching a peak at around POD7-POD14 (Figure 3). On POD7, various types of cells were distributed through the liver tissue compartment, including the portal area, sinusoidal area, and hepatic vein area; however, some came to be located around the portal area, thereafter. CD3^+^ and CD8α^+^ T cells were the major type of accumulating cells, which was consistent with the findings of mass cytometry (Figures 2D and 2F) and remained in the portal area on POD100. I-A^k^-positive cells were another type of portal-infiltrating cells, and they kept locating in the portal area on POD100 (Figure 3). Some I-A^k^-positive cells had CD11c, but Ly6G and F4/80 were almost absent (Figure 3). The expression levels of PD-1 ligands on these I-A^k^-positive cells displayed a notable decrease on POD30 and POD100, whereas the CD206 expression level remained unaltered in the portal vein area (Figures 3 and S2A). Notably most I-A^k^-positive cells of POD14 grafts have not only CD11c and PD-L1 but also PD-L2 and CD206 (Figures 3, S2B, and S2C).

### Temporal and spatial dynamics of T cells

**Figure 4:**
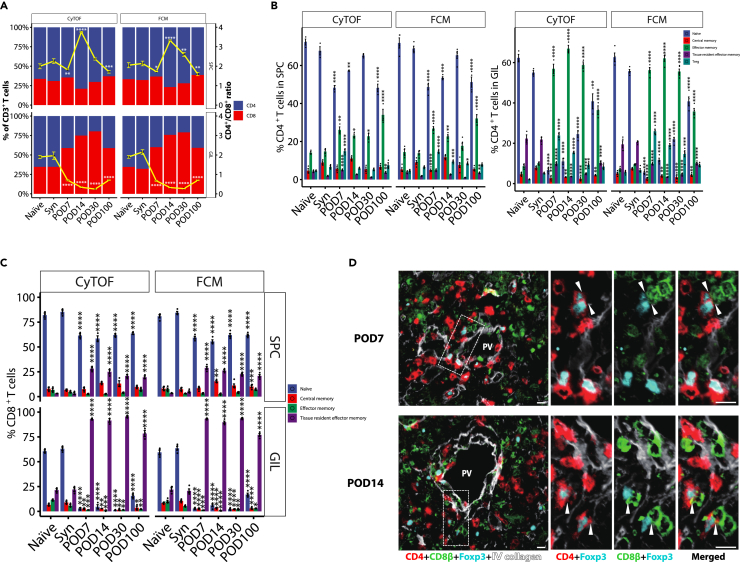
T cell phenotype analysis (A) The proportions of CD4^+^ and CD8^+^ T cell among CD3^+^ T cells and CD4^+^/CD8^+^ T cell ratio, measured by CyTOF and flow cytometry, in spleen cells (SPCs) and graft infiltrating lymphocytes (GILs) of Naïve control (Naïve), syngeneic control (Syn), postoperative days (POD) 7, POD14, POD30 and POD100 groups (n = 3–4 for each group). The yellow dot-line denoted the ratio of CD4^+^/CD8^+^ T cells. ∗p < 0.05, ∗∗p < 0.01, ∗∗∗p < 0.001, and ∗∗∗∗p < 0.0001 by one-way ANOVA with Tukey’s post hoc test vs. syngeneic model control. Values are shown as the mean ± SEM. (B) The proportions of CD4^+^ T cell subsets, measured by CyTOF and flow cytometry, among CD4^+^ T cells of SPCs and GILs in Naïve, Syn, POD7, POD14, POD30, and POD100 groups (n = 3–4 for each group). ∗p < 0.05, ∗∗p < 0.01, ∗∗∗p < 0.001, and ∗∗∗∗p < 0.0001 by one-way ANOVA with Tukey’s post hoc test vs. syngeneic model control. Values are shown as the mean ± SEM. (C) The proportions of CD8^+^ T cell subsets, measured by CyTOF and flow cytometry, among CD8^+^ T cells of SPCs and GILs in Naïve, Syn, POD7, POD14, POD30, and POD100 groups (n = 3–4 for each group). ∗p < 0.05, ∗∗p < 0.01, ∗∗∗p < 0.001, and ∗∗∗∗p < 0.0001 by one-way ANOVA with Tukey’s post hoc test vs. syngeneic model control. Values are shown as the mean ± SEM. (D) Representative immunofluorescence staining of hepatic infiltrating CD4^+^, CD8^+^, and Foxp3^+^ cells on POD7 and POD14. Arrowhead indicated the close association of Foxp3^+^CD4^+^ T cell with CD8^+^ T cell. Image data are representative of three independent experiments. Scale bar = 10 μm.

Since T cells are the major effector immune cell populations involved in transplantation rejection, we further characterized the temporal dynamics of T cells using flow cytometry (FCM). The frequencies of the T cell subclusters in SPCs and GILs were shown in Figures 4A–4C, which were highly comparable between CyTOF and ordinary FCM. The proportion of CD8^+^ T cells in CD3^+^ T cells was larger than 50% in GILs on POD7 and increased over time. The CD4^+^/CD8^+^ ratio remains decreased in the tolerance phase (POD30, 100) in GILs, whereas it showed an opposite trend in SPCs. The effector memory T cells are the major cell population in both CD4^+^ and CD8^+^ T cells in GILs. The proportion of Treg cells peaked on POD7 in both SPCs and GILs and gradually declined over time. Furthermore, many Foxp3^+^CD4^+^ cells were observed around the portal vein area, and some of them were closely associated with CD8β^+^ T cells in the immunofluorescence analysis (Figure 4D).

### T cells exhausted in mouse liver transplantation

**Figure 5:**
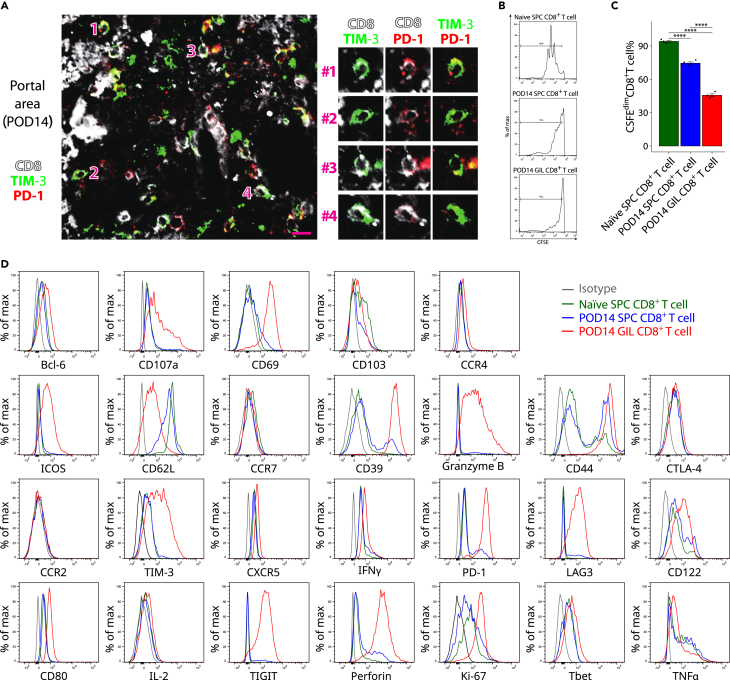
T cell exhaustion in mouse liver transplantation (A) Representative triple immunofluorescence staining of CD8α, PD-1, and TIM3 in hepatic allografts on POD14. Image data are representative of three independent experiments. Scale bar = 20 μm. (B) Representative histogram of CFSE dilution of CD8^+^ T cells in each group (n = 4 for each group). (C) Quantitative data of the proportion of CFSE^dim^ proliferating CD8^+^ T cells in each group (n = 4 for each group). Values are shown as the mean ± SEM. Statistical analysis by one-way ANOVA followed by a post hoc test. ∗∗∗∗p < 0.0001. (D) Representative histogram of molecular markers on CD8^+^ T cells from Naïve SPCs, POD14 SPCs, and POD14 GILs, as evaluated by flow cytometry. Data are representative of four independent experiments.

The PhenoGraph clustering analysis revealed that T cells were the most dominant metaclusters in GILs, accounting for >50% of the GILs in a tolerant mouse liver allograft, and highly expressed PD-1 and lymphocyte activating gene 3 (LAG3) and other markers of exhaustion. We further identified the exhausted CD8^+^ T cells by triple immunofluorescence staining of CD8β, PD-1 and T cell immunoglobulin and mucin-domain containing-3 (TIM3) on POD14 hepatic allografts (Figure 5A). The proliferative capacity of CD8^+^ T cells in POD14 GILs was compromised compared to that in Naïve SPCs and POD14 SPCs, detected by CFSE proliferation assay (Figures 5B and 5C). A flow cytometry analysis of CD8^+^ T cells from Naïve SPCs, POD14 SPCs, and GILs further confirmed that GIL CD8^+^ T cells highly expressed tissue-resident memory and markers of exhaustion, including—but not limited to—PD-1, LAG3, TIM3, and T cell immunoreceptor with Ig and ITIM domains (TIGIT) (Figures 5D and S3). The GIL CD8^+^ T cells also showed the impaired production of proinflammatory cytokines, which was just slightly higher than Naïve SPC CD8^+^ T cells. Although T cell exhaustion was commonly studied in the era of chronic viral infection and antitumor immunity, its role in alloimmune responses remains to be firmly established. The peculiar T cell exhaustion pattern revealed in mouse liver transplantation may provide additional insight into liver tolerance mechanisms.

### CD8^+^CD86^+^ T cell in mouse liver transplantation

**Figure 6:**
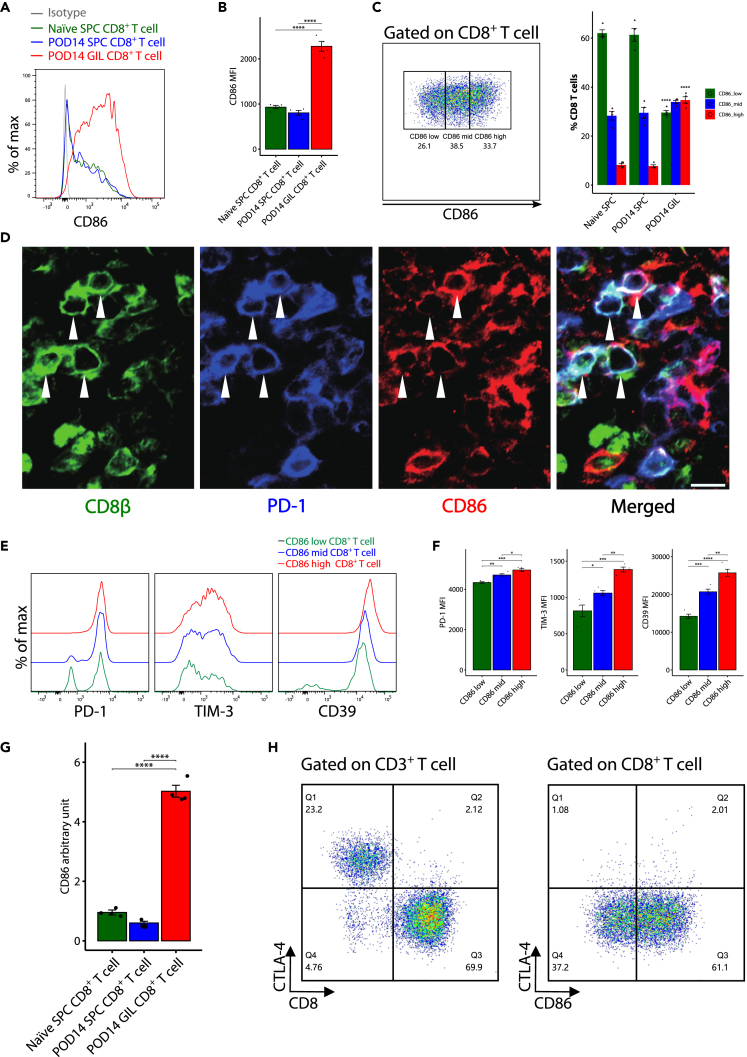
CD86 expression on CD8^+^ T cells in GILs (A) Representative histogram of CD86 expression on CD8^+^ T cells of Naïve spleen cells (SPCs), postoperative day (POD)14 SPCs, and POD14 graft-infiltrating lymphocytes (GILs). Data are representative of four independent experiments. (B) Quantitative data of the median fluorescence intensity (MFI) of CD86 expression on CD8^+^ T cells of Naïve SPCs, POD14 SPCs and POD14 GILs, as evaluated by flow cytometry (n = 4 for each group). Statistical analysis by one-way ANOVA followed by a post hoc test. ∗∗∗∗p < 0.0001. Values are shown as the mean ± SEM. (C) The gating strategy of CD86^low^, CD86^mid^, and CD86^high^ CD8^+^ T cell subsets based on the CD86 expression level (**Left**). The proportion of CD86^low^, CD86^mid^, and CD86^high^ CD8^+^ T cell subsets among CD8^+^ T cells of Naïve SPCs, POD14 SPCs, and POD14 GILs, as evaluated by flow cytometry (n = 4 for each group) (**Right**). ∗∗∗∗p < 0.0001 by one-way ANOVA with Tukey’s post hoc test vs. POD14 SPCs. Values are shown as the mean ± SEM. (D) Representative immunofluorescence staining of CD86, CD8β, and PD-1 in a hepatic allograft on POD14. Image data are representative of three independent experiments. Scale bar = 10 μm. (E) Representative histogram of PD-1, TIM-3, and CD39 expression on CD86^low^, CD86^mid^, and CD86^high^ CD8^+^ T cell subsets of Naïve SPCs, POD14 SPCs, and POD14 GILs. Data are representative of four independent experiments. (F) Quantitative data of the MFI of PD-1, TIM-3, and CD39 expression on CD86^low^, CD86^mid^, and CD86^high^ CD8^+^ T cell subsets of Naïve SPCs, POD14 SPCs, and POD14 GILs, as evaluated by flow cytometry (n = 4 for each group). Statistical analysis by one-way ANOVA followed by a post hoc test. ∗p < 0.05, ∗∗p < 0.01, ∗∗∗p < 0.001, ∗∗∗∗p < 0.0001. Values are shown as the mean ± SEM. (G) The mRNA expression level of CD86 in CD8^+^ T cells of Naïve SPCs, POD14 SPCs, and POD14 GILs. Statistical analysis by one-way ANOVA followed by a post hoc test. ∗∗∗∗p < 0.0001. Values are shown as the mean ± SEM. (H) The representative flow cytometry analysis of CD86 and CTLA-4 expression on CD8^+^ T cells of POD14 GILs. Data are representative of three independent experiments.

Surprisingly, the metacluster C05 T cells also highly expressed costimulatory molecule CD86, which is also known as B7.2 and considered to be mainly expressed on antigen-presenting cells (APCs;e.g., DCs, macrophages, and B cells). The high expression of CD86 on a large proportion of CD8^+^ T cells is an unusual phenomenon. Next, we confirmed the CD86 expression on CD8^+^ T cells by FCM and immunofluorescence analysis. The gating strategy to identify CD8^+^ T cells among the GILs were shown in Figure S4. The median fluorescence intensity of CD86 on CD8^+^ T cells from POD14 GILs was significantly higher than that from POD14 SPCs and Naïve SPCs (Figures 6A and 6B). We further divided CD8^+^ T cells into CD86^low^, CD86^mid^, and CD86^high^ subsets based on the CD86 expression level (Figure 6C). Immunofluorescence staining of liver allografts on POD14 also confirmed that CD86 was expressed on CD8β^+^ T cells and co-expressed with PD-1 (Figure 6D). We found that the expression of CD86 was positively related to the PD-1, TIM-3, and CD39 expression (Figures 6E and 6F), which may indicate that CD86 is a marker of exhaustion.

### Elevated CD86 was expressed by CD8^+^ T cells not by *trans*-endocytosis

CD86 can be captured by CTLA-4 from opposing cells via a process of *trans*-endocytosis.^13^ In order to determine the origin of CD86 on CD8^+^ T cells, we measured the mRNA levels of CD86 in CD8^+^ T cells. As shown in Figure 6G, CD8^+^ T from GILs showed significantly higher mRNA levels of CD86 in comparison to the corresponding counterpart from Naïve or POD14 SPCs. FCM revealed that POD14 GIL CD86^+^CD8^+^ T cells didn’t co-expressed CTLA-4 (Figure 6H). Therefore, the elevated CD86 was expressed by CD8^+^ T cell, not by CTLA-4 mediated *trans*-endocytosis.

## Discussion

In the present study, we depicted the temporal and spatial dynamics of immune cells in mouse liver transplantation by high-dimensional mass cytometry and immunohistochemistry. Our results advanced the understanding of the immune microenvironment dynamics throughout the rejection and acceptance stages after liver transplantation and highlighted several points of importance.

The temporal and spatial kinetics of GILs were investigated by immunohistochemistry. The distribution pattern, as well as the frequency of CD8α^+^ T cells, was quite similar to that of CD3e^+^ T cells, where they appeared throughout the liver tissue on POD7, and subsequently localized at the portal vein area indefinitely. This suggested that most infiltrating T cells were of the CD8 subset and that CD8^+^ T cells that persist at the portal area may play a role in facilitating graft acceptance. In addition, we found that recipient MHC class II (I-A^k^)-positive cells were the second major cell population in graft-infiltrating cells and that their distribution was also localized at the portal vein area. Most I-A^k^-positive cells of POD14 grafts have not only CD11c and PD-L1 but also PD-L2 and CD206. These results suggest that the I-A^k+^CD11C^+^PD-L1^+^PD-L2^+^ cells identified in this study would be identical to CD-DCs, which have been shown to downregulate anti-donor T cell responses *in vitro* and would shed light on the distribution of CD-DCs in tolerant liver grafts.^12^ As the binding affinity of PD-L2 to PD-1 is known to be 3-fold stronger than that of PD-L1,^14^^,^^15^ PD-L2 on CD-DCs may have a critical role in the anti-donor T cell response in tolerance induction. On the other hand, the expression levels of these PD-1 ligands in I-A^k^-positive cells were dramatically decreased on POD30 and POD100, while that of CD206 remained unchanged in the portal vein area. This may suggest that I-A^k^-positive cells other than CD-DCs also play a suppressive role in the maintenance phase.

Alloreactive T cells are the main mediators of transplantation rejection through their direct destruction of allograft cells. Among them, CD8^+^ cytotoxic T cells execute the cell-killing process, whereas CD4^+^ helper T cells coordinate immune responses primarily by secreting cytokines.^16^ A low CD4^+^/CD8^+^ T cell ratio is associated with allograft rejection.^17^ The liver is referred to as the graveyard of T cells, where activated CD8^+^ T cells are eliminated by locally induced apoptosis and the “suicidal emperipolesis” phenomenon.^18^^,^^19^ However, our results revealed that the CD4^+^/CD8^+^ T cell ratio remains low, even in the tolerance phase. Most CD4^+^ or CD8^+^ T cells present in liver allografts were effector memory T cells, which are certainly involved in allograft rejection.^20^^,^^21^ The phenotype of T cells in GILs seems contrary to the spontaneous tolerance of the liver allograft phenomenon. Further analyses revealed that T cells may progress to a functionally defective state, known as exhaustion.

The PhenoGraph clustering analysis revealed that exhausted CD8^+^ T cells (metacluster C05) were the most dominant metacluster in GILs, accounting for more than 50% of the GILs in tolerant liver allografts, and highly expressed PD-1, LAG3, and other exhaustion markers, as well as activation markers. T cell exhaustion is a progressive loss of the effector function that occurs when encountering a persistent high load of antigens or receiving inhibitory signals, which are characteristics of chronic infections and cancer.^22^ With regard to transplantation, alloreactive CD8^+^ T cell exhaustion was also observed following the rapid and extensive initial activation of donor-reactive CD8^+^ T cells early after transplantation in mice, which may contribute to the induction of spontaneous tolerance.^23^^,^^24^ However, the role of T cell exhaustion in spontaneous liver tolerance remains unclear.

We surprisingly found that metacluster C05 T cells highly expressed CD86. CD86 is a central costimulatory molecule that is constitutively expressed on the surface of APCs, which exert critical roles in immune activation. T cells expressing CD86 are less frequently reported in specific pathological conditions.^25^^,^^26^^,^^27^ The function of CD86 expressed on CD8^+^ T cell remains largely unclear. A further FCM analysis revealed that the CD86 expression level was positively associated with the expression of PD-1, TIM-3 and CD39, which may indicate that CD86 is a marker of exhaustion. Such exhausted CD8^+^ T cells persisted in tolerant liver allografts and showed a tissue-resident memory type (CD44^+^CD62L^−^CD69^+^).

Of note, a recent study suggested that activated CD8^+^ T cells expressed the B7 molecule, through which they regulated their own population dynamics via interaction with the CD28 and CTLA-4 receptors on neighboring T cells in a cell count-dependent and cell-density-dependent manner.^28^ The level of CTLA-4 expression in naïve T cells is known to be significantly lower, and it is significantly increased after successful activation. CTLA-4 plays a critical role in the regulation of activated T cells by providing inhibitory effects. Since CD86 has markedly higher affinity for CTLA-4 than CD28, the interactions of CD86 expressed on CD8^+^ T cells with CTLA-4 are expected to be much stronger in comparison to binding with CD28.^29^ Especially in the context of ongoing inflammation, where tissue is densely populated by activated T cells, the CD86 expressed on CD8^+^ T cells may provide critical regulatory signals by interacting with the CTLA-4 expressed on activated T cells. We therefore hypothesized that CD86^+^CD8^+^ T cells play an important role in the induction of liver transplant tolerance. Consistently, the blockade of B7 signaling with CTLA-4Ig could result in impaired T cell regulation, leading to aggressive secondary autoimmunity.^30^^,^^31^

CD86 on CD8^+^ T cells could be endogenously expressed or could be acquired exogenously from opposing cells via a process of *trans*-endocytosis. T cells could capture CD86 molecules from adjacent cells through CTLA-4-mediated *trans*-endocytosis^29^^,^^32^ or TCR-mediated trogocytosis.^33^^,^^34^^,^^35^ However, exogenously captured CD86 molecules would be quickly degraded following internalization, resulting in a lack of (or transient) expression on the cell surface.^32^^,^^36^ In our study, CD86 was persistently expressed on the surface of CD8^+^ T cells. CD8^+^ T cells from GILs also showed significantly higher mRNA levels of CD86 in comparison to Naïve and SPCs CD8^+^ T cells. CD86 was not co-expressed with CTLA-4 on CD8^+^ T cells detected by flow cytometry. In the present study, it is likely that CD86 was endogenously expressed by CD8^+^ T cells in the GILs.

In conclusion, this study provided the first detailed analysis of the temporal and spatial dynamics of immune cells in the mouse liver transplantation model. The temporal and spatial dynamics of immune cells revealed by high-dimensional analyses enable a fine-grained analysis of GIL and SPC subsets, contribute to the discovery of new immunological mechanisms of liver tolerance, and provide potential ways to achieve clinical operational tolerance after liver transplantation.

### Limitations of study

The present study was associated with some limitations. First, due to the antibody panels that we chose, some immune cell lineages could not be detected in our studies (e.g., innate lymphoid cells, basophils, eosinophils, and others). Second, we had only access to several time-points after transplantation. There were no data on the very early temporal and spatial dynamics of immune cells after transplantation. Finally, further investigation of the mechanism and functional validation is necessary to elucidate the role of CD86^+^CD8^+^ T cells in liver tolerance.

## STAR★Methods

### Key resources table

**Table:** 

REAGENT or RESOURCE	SOURCE	IDENTIFIER
**Antibodies**		

Anti-CD45	Fluidigm	3089005C
Anti-Ly-6G	Fluidigm	3141008C
Anti-CD11c	Fluidigm	3142003C
Anti-CD69	Fluidigm	3143004C
Anti-MHC Class I	Fluidigm	3144016C
Anti-CD4	Fluidigm	3145002C
Anti-F4/80	Fluidigm	3146008C
Anti-CD11b (Mac-1)	Fluidigm	3148003C
Anti-CD44	Fluidigm	3150018C
Anti-CD25 (IL-2R)	Fluidigm	3151007C
Anti-CD3e	Fluidigm	3152004C
Anti-CD274 (PD-L1)	Fluidigm	3153016C
Anti-CD152 (CTLA-4)	Fluidigm	3154008C
Anti-CD279 (PD-1)	Fluidigm	3159024C
Anti-CD62L (L-selectin)	Fluidigm	3160008C
Anti-CD40	Fluidigm	3161020C
Anti-Ly-6C	Fluidigm	3162014C
Anti-CD197 (CCR7)	Fluidigm	3164013C
Anti-CD19	Fluidigm	3166015C
Anti-CD335 (NKp46)	Fluidigm	3167008C
Anti-CD8α	Fluidigm	3168003C
Anti-CD206 (MMR)	Fluidigm	3169021C
Anti-CD49b	Fluidigm	3170008C
Anti-CD80 (B7-1)	Fluidigm	3171008C
Anti-CD86	Fluidigm	3172016C
Anti-CD223 (LAG-3)	Fluidigm	3174019C
Anti-B220	Fluidigm	3176002C
Anti-I-A/I-E	Fluidigm	3209006C
Anti-CD16/CD32	BioLegend	101302
Anti-CD3e	Biolegend	100301
Anti-CD4	Biolegend	100426
Anti-CD8α	Biolegend	100701
Anti-CD8β	Biolegend	126627
Anti-CD11c	Biolegend	117301
Anti-CD45R (B220)	BD Biosciences	550286
Anti-CD86	Biolegend	105001
Anti-CD206	Biolegend	141701
Anti-CD273 (PD-L2)	Biolegend	107210
Anti-CD274 (PD-L1)	Biolegend	124301
Anti-CD279 (PD-1)	Biolegend	109111
Anti-CD336 (Tim-3)	Biolegend	119705
Anti-F4/80	Biolegend	123101
Anti-Foxp3	Thermo Fisher Scientific	14-5773-82
Anti-Ly-6G	Biolegend	127601
Anti-I-A^k^	Biolegend	109905
Anti-Type IV collagen	Cosmo Bio	LSL-LB1403
Anti-CD45	Biolegend	103136
Anti-CD3	Biolegend	100328
Anti-CD4	Biolegend	100414
Anti-CD8	Biolegend	100730
Anti-CD86	Biolegend	159204 & 105030
Anti-CD80	Biolegend	104706
Anti-CD69	Biolegend	104512
Anti-PD-1	eBioscience	11-9981-81
Anti-TIM-3	Biolegend	119706
Anti-CD122	eBioscience	17-1222-82
Anti-CCR7	eBioscience	25-1971-82
Anti-CD172a	Biolegend	144006
Anti-PD-L1	Biolegend	124311
Anti-CD39	eBioscience	25-0391-82
Anti-CD103	Biolegend	121416
Anti-CD44	Biolegend	103012
Anti-CD62L	Biolegend	104428
Anti-CXCR5	BD	560577
Anti-TIGIT	Biolegend	142104
Anti-ICOS	Biolegend	313508
Anti-LAG3	Biolegend	125208
Anti-CD107a	Biolegend	121611
Anti-Foxp3	eBioscience	11-5773-82
Anti-Ki-67	eBioscience	25-5698-82
Anti-CTLA-4	eBioscience	17-1522-82
Anti-Perforin	Biolegend	154405
Anti-Granzyme B	Biolegend	372207
Anti-TNF-α	Biolegend	506306
Anti-IL-2	Biolegend	503832
Anti-IFN-γ	Biolegend	505806

**Biological samples**		

Mouse liver transplantation tissues	National Research Institute for Child Health and Development	https://www.ncchd.go.jp

**Chemicals, peptides, and recombinant proteins**		

Cell ID Cisplatin 198Pt	Fluidigm	201198
Fixable Violet Dead Cell Stain Kit	Thermo Fisher Scientific	L34955
Maxpar Cell Staining Buffer	Fluidigm	201068
Formaldehyde Methanol-free	Thermo Fisher Scientific	28906
Cell-ID™ DNA Intercalator-Ir	Fluidigm	201192A
CD8α^+^ T cell Isolation Kit	Miltenyi Biotec	130-095-236
CellTrace CFSE	Thermo Fisher Scientific	C34554
Foxp3 Transcription Factor Staining Buffer Set	eBioscience	00-5523-00
phorbol 12-myristate 13-acetate	Sigma-Aldrich	P1585
ionomycin	Sigma-Aldrich	I9657
Brefeldin A	eBioscience	00-4506-51

**Experimental models: Organisms/strains**		

C57BL/6NCrSlc	Japan SLC, Inc	http://www.jslc.co.jp
C3H/HeNSlc	Japan SLC, Inc	http://www.jslc.co.jp

**Software and algorithms**		

FlowJo software	Tree Star	https://www.flowjo.com/
FIt-SNE	Kluger Lab	https://github.com/KlugerLab/FIt-SNE
PhenoGraph	jacoblevine	https://github.com/jacoblevine/PhenoGraph

**Other**		

Helios mass cytometer	Fluidigm	N/A
LSR Fortessa flow cytometer	BD	N/A

### Resource availability

#### Lead contact

Further information and requests for resources and reagents should be directed to and will be fulfilled by the lead contact, Dr. Xiao-Kang Li (ri-k@ncchd.go.jp).

#### Materials availability

This study did not generate new unique reagents.

### Experimental model and study participant details

#### Animals

Specific pathogen-free inbred male C57BL/6NCrSlc (B6/N; H-2k^b^) and C3H/HeNSlc (C3H; H-2k^k^) mice (age: 8–12 weeks) were purchased from Japan SLC, Inc. (Shizuoka, Japan). All mice received humane care in accordance with the guidelines of the Animal Use and Care Committee of the National Research Institute for Child Health and Development, Tokyo, Japan (Permission number: A2008-004-C10). All mouse experiments conformed to the National Institutes of Health guidelines for the care and use of laboratory animals.

#### Mouse liver transplantation

Orthotopic mouse liver transplantation was performed using a combined cuff and suture technique without hepatic artery reconstruction under inhalation anesthesia as described in detail, without immunosuppressive therapy.^10^

### Method details

#### Mass cytometry

Cells were stained with Cell-ID Cisplatin-198Pt (Fluidigm, South San Francisco, CA; Cat#201198) for 5 min at a final concentration of 1 μM at room temperature to differentiate live vs. dead populations, then incubated with FcγR-blocking anti-CD16/CD32 (BioLegend, San Diego, CA; Cat#101302) for 10 min at room temperature to prevent non-specific antibody binding. Cells were then washed twice with Maxpar Cell Staining Buffer (Fluidigm; Cat#201068), and then stained with a master mix containing different metal-labeled antibodies (Table 1) diluted in Maxpar Cell Staining Bufferfor 30 min at room temperature. After staining, cells were washed two times with cell-staining media and then fixed with 4% v/v paraformaldehyde at 4°C overnight. Cells were finally stained with Cell-ID DNA Intercalator-Ir (Fluidigm; Cat#201192A) and washed. Cells were washed and resuspended in MilliQ water, before being acquired on a Helios mass cytometer (Fluidigm).

#### Immunohistochemistry

Double immunoenzyme staining was performed as described previously.^37^ In brief, 4-μm-thick fresh cryosections of hepatic allograft were incubated first with the antibody for the target cell (alkaline phosphatase-blue), then for type IV collagen (peroxidase-brown). In some cases, formalin-fixed, paraffin-embedded sections were prepared.

Four-color immunofluorescence consisting of three-color staining for target cells and one for type IV collagen (Cosmo Bio; Cat#LSL-LB1403), was performed according to a previously reported procedure, with minor modification.^37^ For three target cell staining, indirect staining with anti-target cell antibodies raised in rat and Alexa Fluor 594 conjugated anti-rat IgG was performed first. After washing more than three times with PBS-0.2% Tween 20, the section was incubated with 30 μg/mL of normal rat IgG (Jackson Immuno Research, West Grove, PA) to block the redundant antigen binding site in anti-rat IgG that was used in the first staining procedure. Next, the slide was incubated with two monoclonal antibodies directly coupled to different fluorochromes, such as Alexa Fluor 488, allophycocyanin. Finally, type IV collagen staining was performed. The antibodies used for Immunohistochemistry studies listed in Table S1. Multichannel color fluorescence images were captured using an Axioskop 2 Plus fluorescence microscope equipped with an AxioCam MRm camera (Zeiss, Oberkochen, Germany). We assigned pseudocolors to each channel to create merged images that were more comprehensible by maximizing contrast using the AxioVision software program (Zeiss).

#### Isolation of mouse liver graft infiltrating lymphocytes (GILs)

Mouse liver GILs were isolated by mechanical disruption and centrifugal elutriation as described previously,^38^ with minor modifications. Briefly, liver grafts were perfused with 2 mL of phosphate-buffered saline (PBS) via the portal vein to remove blood and harvested at the indicated timepoints. Next, liver grafts were minced into small pieces using surgical scissors, gently passed through a 100 μm gauge mesh using a sterile syringe plunger and suspended in 50 mL of PBS containing 10% fetal calf serum (Thermo Fisher Scientific, Waltham, MA). The cell suspension was centrifuged without brake at 60 ×*g* for 1 min at room temperature to remove hepatocytes, and then washed two times at 500 ×*g* for 8 min at room temperature. The collected cell pellet was resuspended in 10 mL of 33% Percoll (GE Healthcare, Piscataway, NJ), and then centrifuged without brake at 1000 ×*g* for 20 min at room temperature. The GILs were finally obtained after erythrocyte lysing using ammonium chloride solution and subsequently subjected to flow cytometry or mass cytometry.

#### CyTOF data analysis

After acquisition, raw data were debarcoded using a doublet-filtering scheme^39^ with unique mass-tagged barcodes. Batch effects were normalized through bead normalization method.^40^ Data were manually gated to exclude to debris, dead cells and doublets, leaving live, single immune cells using a FlowJo software (version 10, Tree Star, San Carlos, CA). Signal intensities for each channel were arcsinh transformed with a cofactor of 5 (x_transf = asinh(x/5)). Dimensionality reduction algorithm Fast interpolation-based t-SNE^41^ was performed to visualize the high-dimensional data in two dimensions and show distribution of each cluster and marker expression and difference among each group or different sample type. Phenograph clustering algorithm^42^ (*k* = 30, Resolution = 0.5) was applied to identify the major cell subset based on marker expression levels. Cell type of each cluster was annotated according to its marker expression pattern on a heatmap of cluster vs. marker.

#### CD8^+^T cell isolation

CD8^+^ T cells were purified from the Naive SPCs and POD14 SPCs and POD14 GILs using magnetic-activated cell sorting (MACS) mouse CD8^+^ (Miltenyi Biotec, Bergisch Gladbach, Germany; Cat#130-095-236) T cell Isolation Kits according to the manufacturer’s instructions.

#### CFSE proliferation assay

In the CFSE proliferation assay, MACS-isolated CD8^+^ T cells from the Naive SPCs and POD14 SPCs and POD14 GILs were labeled with CellTrace CFSE (Thermo Fisher Scientific, Cat# C34554). The CD8^+^ T cells were stimulated with plate-bound anti-CD3 (2 μg/mL) and anti-CD28 (2 μg/mL) in a 96-well flat-bottom plate (1 × 10^5^ per well) with 100 μL per well of complete RPMI 1640 medium at 37°C for 3 days. At the end of the assay, cells were collected for measurement of CFSE dilution by flow cytometry.

#### Flow cytometry

Cells were stained with a Fixable Violet Dead Cell Stain Kit (Thermo Fisher Scientific; Cat# L34955) for 30 min at 4°C to label dead cells, then incubated with FcγR-blocking anti-CD16/CD32 for 10 min at room temperature to prevent non-specific antibody binding. For cell surface staining, the cells were incubated for 30 min at 4°C with different combinations of fluorochrome-conjugated antibodies against mouse CD45 (BioLegend; Cat#103136), CD3 (BioLegend; Cat#100328), CD4 (BioLegend; Cat#100414), CD8 (BioLegend; Cat#100730), CD86 (BioLegend; Cat#159204 & 105030), CD80 (BioLegend; Cat#104706), CD69 (BioLegend; Cat#104512), PD-1 (eBioscience, San Diego, CA; Cat#11-9981-81), TIM-3 (BioLegend; Cat#119706), CD122 (eBioscience; Cat#17-1222-82), CCR7 (eBioscience; Cat#25-1971-82), CD172a (BioLegend; Cat#144006), PD-L1 (BioLegend; Cat#124311), CD39 (eBioscience; Cat#25-0391-82), CD103 (BioLegend; Cat#121416), CD44 (BioLegend; Cat#103012), CD62L (BioLegend; Cat#104428), CXCR5 (BD Biosciences, Franklin Lakes, NJ; Cat#560577), TIGIT (BioLegend; Cat#142104), ICOS (BioLegend; Cat#313508), LAG3 (BioLegend; Cat#125208), CD107a (BioLegend; Cat#121611). For intracellular staining, cells were fixed and permeabilized using Foxp3 Transcription Factor Staining Buffer Set (eBioscience; Cat#00-5523-00) and then stained with the following fluorochrome-conjugated antibodies against: Foxp3 (eBioscience; Cat#11-5773-82), Ki-67 (eBioscience; Cat#25-5698-82), CTLA-4 (eBioscience; Cat#17-1522-82), Perforin (BioLegend; Cat# 154405), Granzyme B (BioLegend; Cat#372207), TNF-α (BioLegend; Cat#506306), IL-2 (BioLegend; Cat#503832), IFN-γ (BioLegend; Cat#505806). For intracellular cytokine staining, cells were stimulated with 50 ng/mL phorbol 12-myristate 13-acetate (Sigma-Aldrich, St. Louis, MO; Cat#P1585), 1 μg/mL ionomycin (Sigma-Aldrich; Cat#I9657) and Brefeldin A (eBioscience; Cat#00-4506-51) in complete medium for 4 h, followed by surface and intracellular staining. Appropriate fluorochrome-conjugated, isotype-matched IgG was used as a negative control. Flow data were acquired on an LSR Fortessa flow cytometer (BD Biosciences) and analyzed using a FlowJo software program.

#### Histological analyses

Hepatic allografts were fixed in 10% neutral buffered formalin (Wako, Osaka, Japan; Cat#060–01667), dehydrated, and then embedded in paraffin. Paraffin sections (4-μm-thick) were stained with hematoxylin and eosin stain (H&E). Hepatic allograft rejection activity index (RAI) was graded by two investigators blinded to the group and labeling allocation during the experiment, according to the World Gastroenterology Consensus Document criteria.^43^

### Quantification and statistical analysis

All statistical analyses were performed using GraphPad Prism (version 7.00; GraphPad Software Inc., San Diego, CA). The results are expressed as the mean ± standard error of the mean (SEM). Student’s *t* test (normal distribution data) was used for comparisons between two groups. Multiple comparisons on a single dataset were performed by a one-way analysis of variance (ANOVA) followed by a post hoc test. A log rank (Mantel-Cox) test was used for the survival data. In all experiments, differences were considered statistically significant at ∗p < 0.05, ∗∗p < 0.01, ∗∗∗p < 0.001, and ∗∗∗∗p < 0.0001.

### Additional resources

We have no relevant resources.

## Data Availability

• Data: All the data reported in this study will be shared by the lead contact upon request. • Code: This paper does not report original code. • Additional information: Any additional information required to reanalyze the data reported in this paper is available from the lead contact upon request.
