# Supplementary material for: Temporal and spatial dynamics of immune cells in spontaneous liver transplant tolerance
Source: iScience. 2023 Aug 19;26(9):107691. doi: 10.1016/j.isci.2023.107691 (PMC10485166; doi:10.1016/j.isci.2023.107691)
Supplement: Document S1. Figures S1–S4 and Table S1 [file mmc1.pdf]

iScience, Volume *26*

Supplemental information

Temporal and spatial dynamics of immune cells in spontaneous liver transplant tolerance

Weitao Que, Hisashi Ueta, Xin Hu, Miwa Morita-Nakagawa, Masayuki Fujino, Daisuke Ueda, Nobuko Tokuda, Wenxin Huang, Wen-Zhi Guo, Lin Zhong, and Xiao-Kang Li

Supplementary Materials

Fig. S1. ***The phenotype of rejection activity after liver transplantation.*** **Related to Figure 1.** The rejection activity index in Naïve control (Naïve), syngeneic control (Syn), postoperative days (POD) 7, POD14, POD30 and POD100 groups (n = 6 for each group). Values are shown as the mean ± SEM.

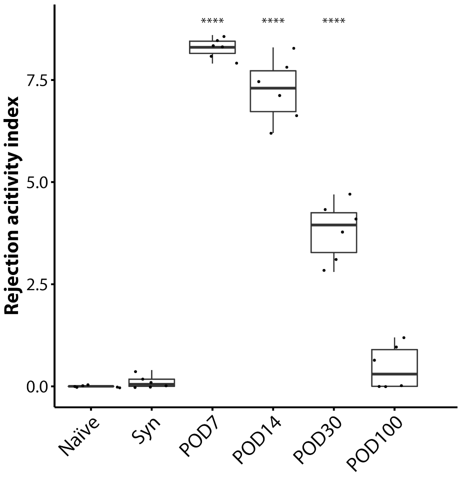

Fig. S2. ***I-A^{k+}CD11C^{+}PD-L1^{+}PD-L2^{+} cells in mouse liver transplantation.*** **Related to Figure 3.** (**a**) Representative double immunoenzyme staining of PD-L1, PD-L2 and CD206 in hepatic grafts in Naïve control (Naïve), postoperative days (POD) 7, POD14, POD30 and POD100 groups. Image data are representative of three independent experiments. Scale bar = 200 µm. (**b**) Representative immunofluorescence staining of CD11c, PD-L1 and PD-L2 in a hepatic allograft on POD14. Image data are representative of three independent experiments. Left scale bar = 100 µm, right scale bar = 20 µm. (**c**) Representative immunofluorescence staining of CD11c, CD206 and PD-L2 in a hepatic allograft on POD14. Image data are representative of three independent experiments. Left scale bar = 100 µm, right scale bar = 20 µm.

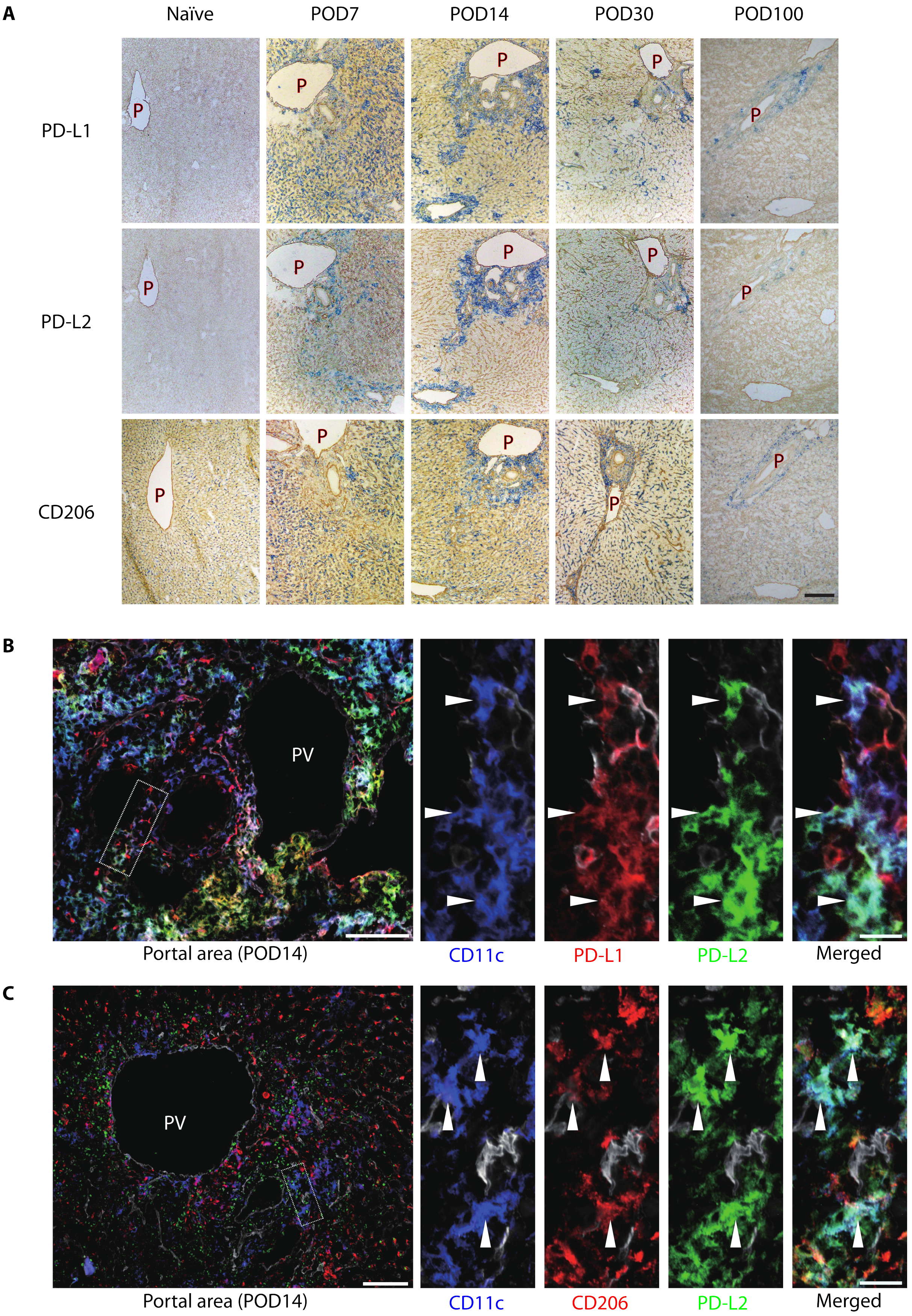
A
Naïve
POD7
POD14
POD30
POD100
PD-L1
PD-L2
CD206
P
B
PV
Portal area (POD14)
CD11c
PD-L1
PD-L2
Merged
C
PV
Portal area (POD14)
CD11c
CD206
PD-L2
Merged

Fig. S3. ***T cell exhaustion in mouse liver transplantation.*** **Related to Figure 5.** Quantitative data of the median fluorescence intensity (MFI) of molecule markers on CD8^{+} T cells from Naïve SPCs, POD14 SPCs and POD14 GILs, as evaluated by flow cytometry. (n = 4 for each group). Values are shown as the mean ± SEM. Statistical analysis by one-way ANOVA followed by a post hoc test. ns, not significant, *: p<0.05, **: p<0.01, ***: p<0.001, ****: p<0.0001.

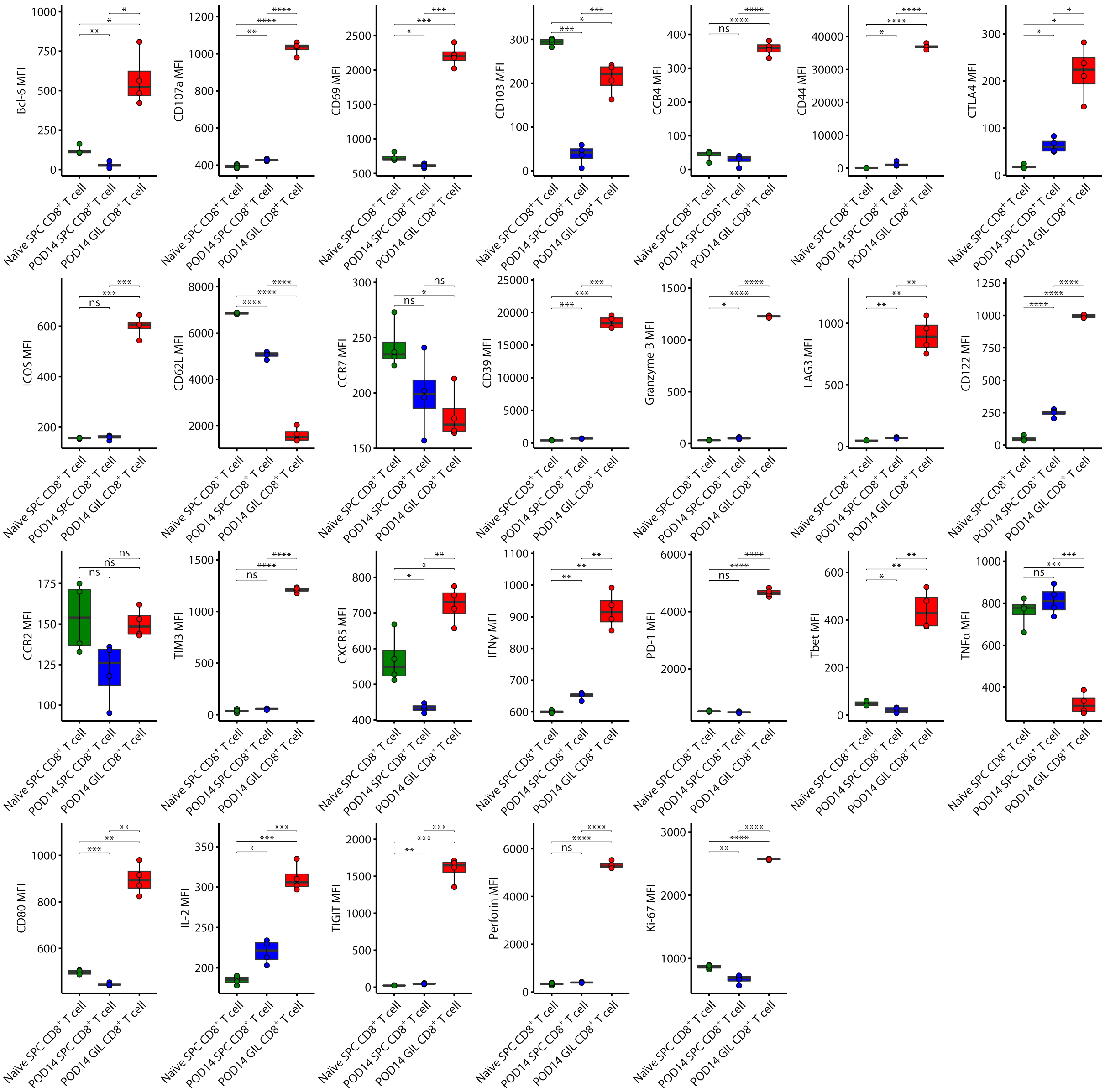

Fig. S4. ***The gating strategy to identify CD8^{+} T cells among the GILs*. Related to Figure 6.** Total events were gated for single cells, and then for living leukocytes. CD8^{+} T cell were gated as CD3, CD8 double positive populations.

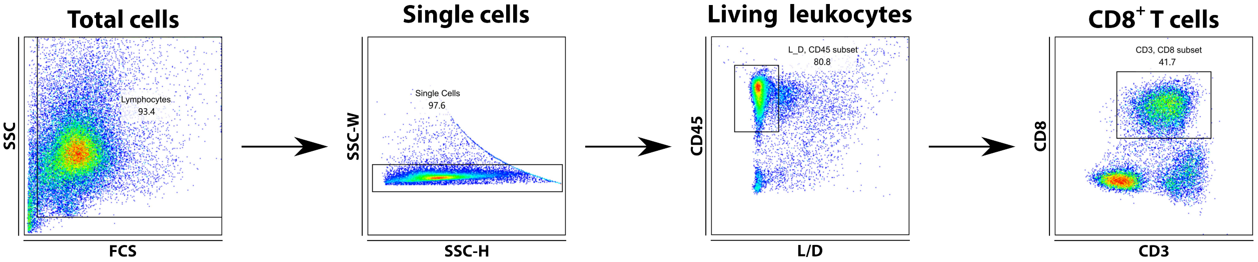

Table S1. List of antibodies used for immunohistochemistry. Related to STAR Methods.

Anti-bodies	Source	Catalog number
Anti-CD3e	100301	Biolegend
Anti-CD4	100426	Biolegend
Anti-CD8α	100701	Biolegend
Anti-CD8β	126627	Biolegend
Anti-CD11c	117301	Biolegend
Anti-CD45R (B220)	550286	BD Biosciences
Anti-CD86	105001	Biolegend
Anti-CD206	141701	Biolegend
Anti-CD273 (PD-L2)	107210	Biolegend
Anti-CD274 (PD-L1)	124301	Biolegend
Anti-CD279 (PD-1)	109111	Biolegend
Anti-CD336 (Tim-3)	119705	Biolegend
Anti-F4/80	123101	Biolegend
Anti-Foxp3	14-5773-82	Thermo Fisher Scientific
Anti-Ly-6G	127601	Biolegend
Anti-I-A^k	109905	Biolegend
Anti-Type IV collagen	LSL-LB1403	Cosmo Bio
